# Higher-order G-quadruplexes in promoters are untapped drug targets

**DOI:** 10.3389/fchem.2023.1211512

**Published:** 2023-06-07

**Authors:** Robert C. Monsen

**Affiliations:** Department of Medical Oncology and Hematology, UofL Health Brown Cancer Center, University of Louisville, Louisville, KY, United States

**Keywords:** G-quadruplex (G4), higher-order G4, DNA, promoter, drug target

## Abstract

G-quadruplexes (G4s) are four-stranded nucleic acid secondary structures that form within guanine-rich regions of chromatin. G4 motifs are abundant in the genome, with a sizable proportion (∼40%) existing within gene promoter regions. G4s are proven epigenetic features that decorate the promoter landscape as binding centers for transcription factors. Stabilizing or disrupting promoter G4s can directly influence adjacent gene transcription, making G4s attractive as indirect drug targets for hard-to-target proteins, particularly in cancer. However, no G4 ligands have progressed through clinical trials, mostly owing to off targeting effects. A major hurdle in G4 drug discovery is the lack of distinctiveness of the small monomeric G4 structures currently used as receptors. This mini review describes and contrasts monomeric and higher-order G-quadruplex structure and function and provides a rationale for switching focus to the higher-order forms as selective molecular targets. The human telomerase reverse transcriptase (hTERT) core promoter G-quadruplex is then used as a case study that highlights the potential for higher-order G4s as selective indirect inhibitors of hard-to-target proteins in cancer.

## 1 G-quadruplex structures in promoters: monomeric vs. higher-order forms

### 1.1 Intramolecular monomeric promoter G-quadruplexes

**FIGURE 1:**
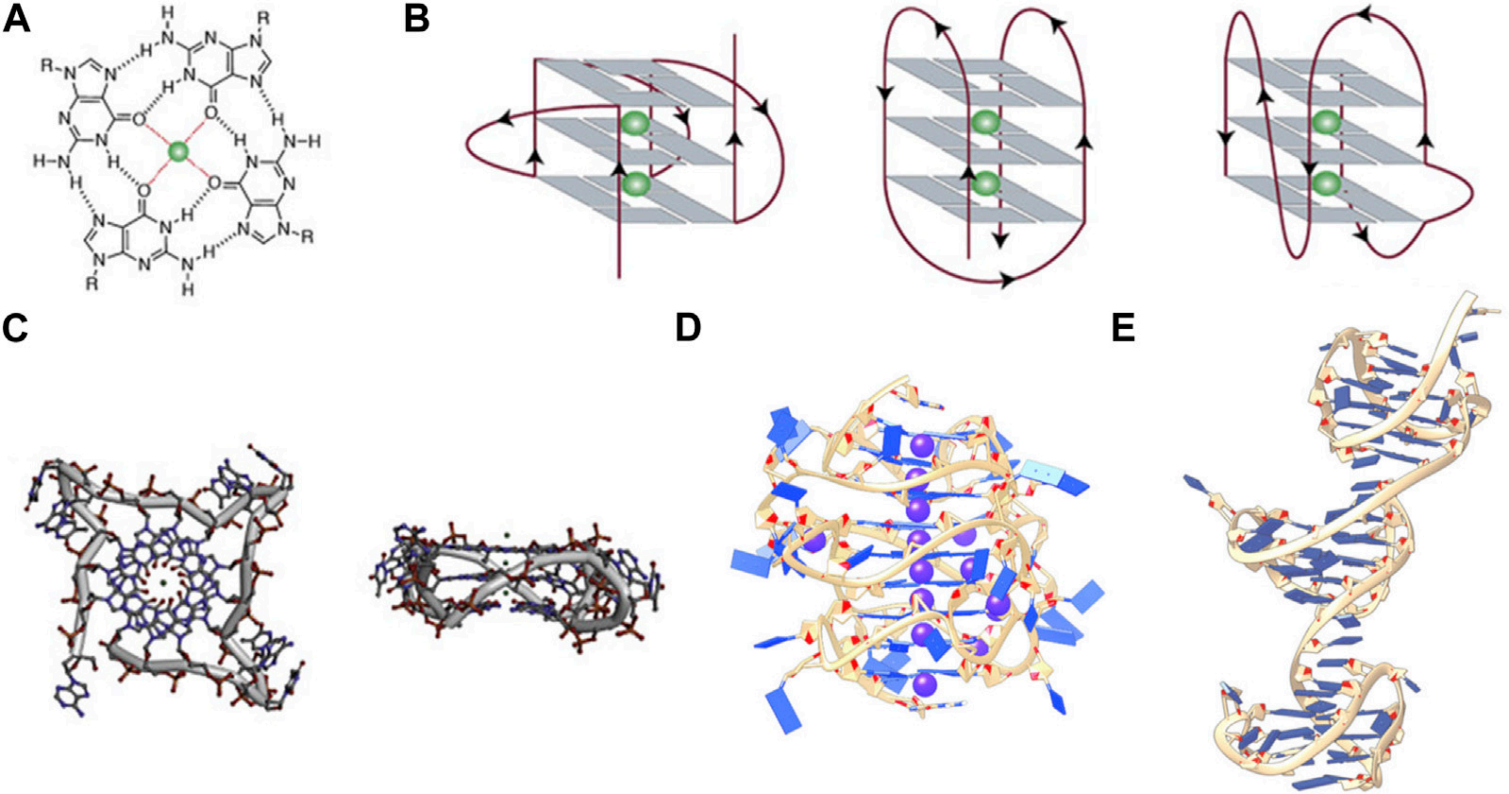
**(A)** Structure of a G-tetrad, **(B)** From left to right, schematic representations of **(A)** parallel, antiparallel, and mixed hybrid with bulge, **(C)** Top and side views of a parallel monomeric G-quadruplex, **(D)** side view of a stacked intramolecular all-parallel higher-order G-quadruplex, and **(E)** side view of an intramolecular higher-order all-hybrid G-quadruplex in a beads-on-a-string arrangement. **(C–E)** are not to scale. Figures 1A–C, are adapted from (Spiegel et al., 2020), **(D)** is adapted from (Monsen et al., 2020), and **(C)** is adapted from (Monsen et al., 2021).

G-quadruplexes (G4s) are four-stranded secondary structures created from the stacking of two or more guanine tetrads (“G-tetrads”) (Spiegel et al., 2020). Each G-tetrad is composed of four guanine bases arranged in a square planar configuration, that is, stabilized by Hoogsteen hydrogen bonding (Figure 1A). Monovalent cations are coordinated within the G-tetrad column central channel by the inward facing carbonyl groups, providing stabilization from coordinate bonding and neutralization of the partially negative charges (Lane et al., 2008). A commonly used sequence motif to describe a monomeric G-quadruplex is G_3-4_L_1-7_G_3-4_L_1-7_G_3-4_L_1-7_G_3-4_, where G indicates a guanine tract and L designates any nucleotide in the intervening loop. Historically, the largest loop length has been taken to be seven nucleotides because of the destabilizing effect of large loops *in vitro* (Spiegel et al., 2020; Ravichandran et al., 2021).

G-rich sequences in specific gene promoter regions can adopt G4 structures with a surprisingly diverse array of topological conformations for the intramolecular monomeric G4 *in vitro*, depending on the sequence composition, loop lengths, ionic environment, and hydration (Chen and Yang, 2012; Miller et al., 2010; Bhattacharyya et al., 2016). The most frequent topologies are: antiparallel, in which two out of four G-tracts run in the same direction; hybrid, in which three G-tracts run in the same direction; and parallel, in which all G-tracts run in the same direction (Figure 1B) (Spiegel et al., 2020). This review is primarily concerned with promoter G4s that are necessarily intramolecular and flanked on either end by single- or double-stranded regions. In the context of a promoter bubble (Shirude et al., 2007; Monsen et al., 2023), as well as when promoter G4 sequences are flanked by single-stranded regions (this can be realized *in vitro,* for example, by adding two residues on either end of the monomer G4 motif: L_2_G_x_L_n_G_x_L_n_G_x_L_n_G_x_L_2_), the parallel topology is preferred (Chen et al., 2021). The biological preference for parallel promoter G4 conformations is supported by the extensive immunofluorescence seen across chromosomes stained with the parallel-favoring anti-G4 antibody “BG4” (Biffi et al., 2013; Javadekar et al., 2020). The first ever near-atomic structural study of a G4 in a near-native duplex bubble has shown that duplex end-stacking at the G-tetrad interface may be a key factor to promoter G4 stability, suggesting that antiparallel and hybrid topologies could be sterically prohibited (Monsen et al., 2023). Overall, *in vitro* monomeric promoter G4 sequences adopt a variety of topological configurations when isolated, but when placed in their biological context prefer the parallel form.

### 1.2 Intramolecular higher-order promoter G-quadruplexes

Intramolecular higher-order promoter G4s (xG4s) consist of two or more connected monomeric G4 domains. The xG4 motif is degenerate compared to the lower order G4 motif and is of the general form (G_2-4_L_1-12_G_2-4_L_1-12_G_2-4_L_1-12_G_2-4_L_≥0_)_n_ (Monsen et al., 2022a), where the length of the 3′ connecting loop can be zero and n ≥ 2. The xG4 motif becomes substantially more degenerate when accounting for mismatches and/or bulges (i.e., non-guanine nucleotides within a putative G-tract) (Berselli et al., 2020). The xG4 motif is notable for multiple reasons. First, the zero-nucleotide connecting loop presents the possible situation where the last guanine of the 3′ G-tetrad of the first G4 domain and the first guanine of the 5′ G-tetrad of the second G4 domain create a continuous G-tetrad column that spans the entire macromolecule. If both G4s are parallel, this would facilitate formation of a single continuous medium groove spanning the two domains with a favorable locked 3′ to 5′ G-tetrad stacked interface (Kogut et al., 2019). A second, less obvious feature of the xG4 motif is that it accounts for two-tetrad G4s, in which there are only two guanines in the G-tracts. Two-tetrad monomeric G4s cannot exist as stable parallel conformations *in vitro* when isolated (Kejnovska et al., 2021), but have been seen in higher-order G4 assemblies (Monsen et al., 2022a) [we note that stable antiparallel two-tetrad G4s are seen *in vitro* (Lim et al., 2009)]. This is an important feature, as it reveals that biologically relevant xG4s have an expanded structural repertoire compared to the monomeric sequences. Lastly, xG4s seem to tolerate much longer loops (1-12+ nucleotides), both within and between the G4 domains (Berselli et al., 2020; Monsen et al., 2020; Monsen et al., 2022a). This may be the result of tertiary interactions that stabilize either across the G4-G4 domain by loop interactions (Rigo and Sissi, 2017), symmetry in the loop giving rise to a stabilizing hairpin moiety (Berselli et al., 2020), or arise from the stability imparted on the G4 cores from head-to-tail stacking (Kogut et al., 2019).

Structural characterizations of intramolecular promoter xG4s to date have revealed an overwhelming preference for stacked arrangements (Micheli et al., 2010; Rigo and Sissi, 2017; Monsen et al., 2020; Monsen et al., 2022a), with the highest resolution models showing a preference for an all-parallel stacked globular configuration (Figure 1D) (Monsen et al., 2020; Monsen et al., 2022a). Importantly, all reported putative xG4 sequences that have been investigated form stable G4 structures *in vitro*. In some cases, the presence of long loops (>7 nucleotides) result in hairpins (Monsen et al., 2022a) that contribute to the stability of the individual domains (Ravichandran et al., 2021) while also creating structurally unique interfaces. In contrast to promoter xG4s, other higher-order intramolecular G4s reported, such as the human telomere (Monsen et al., 2021), the insulin-linked polymorphic region (ILPR) minisatellite (Schonhoft et al., 2009), and the CEB25 minisatellite locus (Amrane et al., 2012), show mixed antiparallel and hybrid topologies that are better described as dumbbell shaped or beads-on-a-string configurations. Figure 1 contrasts the structures of a monomer G4 in the parallel conformation, a higher-order all-parallel stacked G4, and a higher-order all-hybrid beads-on-a-string.

The selective binding interface of the xG4s is not imparted by the G-tetrad columns, but rather the specific G4-G4 interaction interfaces, loop sequence, size, and configurations. Figure 1C reveals that there is little targetable real estate associated with the monomeric parallel G4 (Monsen et al., 2023). Conversely, Figures 1D, E reveals multiple putative binding sites that would be large enough and distinct enough for specific protein or drug interaction. Biochemical support for this idea comes from a recent pull-down study conducted by Ceschi et al. (Ceschi et al., 2022). In this study, the authors used a variety of higher-order G4 sequences (such as those in Figures 1D, E) to enrich for tightly interacting proteins from cell lysates that are specific to higher-order G4s over the lower order forms. Surprisingly, the intermediate filament Vimentin was shown to have a selective nanomolar affinity to higher-order G4 structures with no apparent binding to lower order species. While the mechanism of recognition is still unclear, this study supports the hypothesis that xG4s offer unique recognition sites that could be useful in selective targeting.

## 2 G-quadruplex distribution and function in promoters

### 2.1 Biological distribution and function of promoter G4s

Promoter G-quadruplexes are prevalent epigenetic regulatory elements. Current estimates show more than 700,000 monomeric G4 motifs across the human genome (Hansel-Hertsch et al., 2017). Huppert and Balasaubramanian, using the canonical sequence motif G_3+_L_1-7_G_3+_L_1-7_G_3+_L_1-7_G_3+_, showed that more than 40% of gene promoters have at least one monomeric G4 motif (Huppert and Balasubramanian, 2007). More recently, Hänsel-Hertsch and colleagues have used a chromatin immunoprecipitation (ChIP)-sequencing direct capture approach to show that thousands of G4 structures, not just motifs, are enriched in highly transcribed gene promoters, specifically many involved in cancer (Hansel-Hertsch et al., 2016). Promoter quadruplexes overall appear to be acting as general transcription factor (TF) “binding hubs”, coinciding with regions of open chromatin and high transcriptional activity (Spiegel et al., 2021).

At a more granular level, promoter G4s act in concert with transcriptional proteins to affect gene transcription in multiple ways. Initially, promoter G4s were thought to only act as simple physical barriers to polymerases, acting as “on/off” switches of transcription (Sarkies et al., 2010). However, studies have now shown that G4s can directly recruit transcription factors with some level of specificity. For instance, two zinc fingers, SP1 and MAZ (Myc-associated zinc finger), show G4 structure-dependent recognition. In the former case, Raiber and colleagues, using pull-down experiments with the transcription factor SP1, showed that 36% of the sequences lacked consensus SP1 binding motifs (Raiber et al., 2012). They went on to show that 77% of those sequences lacking the SP1 consensus motif were putative G-quadruplexes and that, overall, SP1 binding had 87% overlap with G4 sequence motifs. In the latter case, Cogoi and colleagues have shown that MAZ recognizes a G4 formed within the *kRas* promoter and showed that stabilizing the *kRas* G4 with a small molecule could promote MAZ binding and increase transcription, while mutations that destabilized the G4 reduced MAZ binding and transcription (Cogoi et al., 2010).

G4s can also serve as transcriptional repressors. The classical case for this is the G4 formed within the *c-Myc* promoter. In their seminal study, Siddiqui-Jain et al. showed that a G-rich region in the nuclease hypersensitivity element III (NHEIII) of the c-Myc P1 promoter forms one or more monomeric G4s (Siddiqui-Jain et al., 2002). Through mutational destabilization, they showed that there is a 3-fold increase in c-Myc expression, indicating that the G4 acts as a transcriptional repressor. They showed that stabilization of the G4 with a small molecule could further reduce transcription to below the basal level. Later studies on the protein nucleolin, a multifaceted and abundant protein found in the nucleolus (Tajrishi et al., 2011), have shown that it can help the folding of promoter G4s like a molecular chaperone (Tosoni et al., 2015). Nucleolin was shown to fold the c-Myc NHEIII G4, promoting transcriptional downregulation in cells (Gonzalez and Hurley, 2010). Altogether, these studies show that promoter G4s are important epigenetic regulators of genes, and that their stability and interaction with transcription factors can influence transcription.

### 2.2 Biological distribution and function of promoter xG4s

Promoter xG4s are also abundant across the genome. Berselli and colleagues recently developed QPARSE (13), the first algorithm capable of finding xG4s in the genome that accounts for mismatches and bulges in G-tracts (see Figure 1B for a G4 with a bulge). In the study, the authors used their degenerate G4 motif algorithm to find monomeric, dimeric, and trimeric G4 sequences in the range of −200 to +600 of the transcriptional start sites (TSS) across the annotated human genome [GENCODE (Harrow et al., 2012)]. They found that 49%, 15%, and 4% of TSS regions had monomer, dimer, and trimer G4 repeats, respectively, and show that this enrichment cannot entirely be attributed to high GC content. Ceschi et al. have recently used the same algorithm to search just the first 100 bp upstream of the TSS of gene promoters in GENCODE, finding 1,478 dimer and trimer promoter xG4s (Ceschi et al., 2022) (∼4% of the 38,404 annotated genes in GENCODE v34).

Parsing out the *in vivo* function of a particular xG4 compared to their monomeric counterparts is a challenging task. At one end you have bioinformatic and G4-or G4-ChIP-sequencing approaches that lack the spatial resolution to distinguish between monomer, dimer, trimer, and other higher-order G4 effects (Park, 2009; Mahony and Pugh, 2015). For instance, the ChIP-sequencing studies mentioned above should, in theory, encompass promoter sites enriched with xG4s. At the other end, there is a dearth of tools that allow direct probing of the effects of monomer versus higher-order G4s. Specifically, there are no reports to date that have convincingly shown selective (de)stabilization of a single G4 domain among a higher-order promoter G4 assembly (Frasson et al., 2022). Mutational reporter assays are the current best approach to parsing out the functionality of xG4s within a cellular context (Siddiqui-Jain et al., 2002; Cogoi et al., 2010), but these come at the risk of altering protein recognition motifs (Bell et al., 2015).

The most thorough investigation of a promoter xG4 to date was conducted in 2019 by Ducani and colleagues on an xG4 found in the promoter of the proto-oncogene *c-Kit* (Ducani et al., 2019). The *c-Kit* gene encodes for a transmembrane tyrosine kinase receptor (c-Kit or CD117) that, after activation by stem cell factor (SCF), transduces signals that promote cell proliferation, differentiation, and migration (Liang et al., 2013). Excessive signaling by continued stimulation or mutation has been implicated in a variety of cancers, such as gastrointestinal stromal tumors (GISTs), pancreatic cancer, melanoma, and hematological neoplastic diseases (Gregory-Bryson et al., 2010; Abbaspour Babaei et al., 2016). Since c-Kit has a tyrosine kinase domain, these cancers are typically treated with tyrosine kinase inhibitors (TKIs) tailored to the mutation type (Abbaspour Babaei et al., 2016). Unfortunately, treating c-Kit-driven cancers with TKIs is often followed with a rapid switch to drug-resistance through mutation (Demetri et al., 2002; Loughrey et al., 2006; Abbaspour Babaei et al., 2016). Therefore, understanding how the *c-Kit* promoter xG4 regulates its transcription is important for drug development efforts. The c-Kit xG4 consists of three monomeric G4 motifs, designated K2, SP, and K1 (from 5′ to 3′) connected by a single dA loop and a hexanucleotide dGCGCAG loop, respectively. Each separate G4 domain has been structurally and/or functionally examined (Rankin et al., 2005; Phan et al., 2007; Hsu et al., 2009; Kuryavyi et al., 2010; Raiber et al., 2012; Kotar et al., 2019), and the higher-order structural assembly of domains K2-SP confirmed by integrative structural biology approaches (Rigo and Sissi, 2017; Monsen et al., 2022a). Both structural studies show that the K2-SP regions interact through stacking, although the two studies differ slightly in the size of the sequence examined. Based on circular dichroism of the full-length sequence used by Ducani, however, the full-length K2-SP-K1 sequence adopts an all-parallel conformation, which is consistent with the promoter xG4s structurally verified to date (Monsen et al., 2020; Monsen et al., 2022a). To investigate the biological function of such an arrangement, Ducani and colleagues conducted luciferase assays to test the effect of disrupting all combinations of the G4 regions by mutating guanines essential for structural integrity. They show in the leukemia cell line HEL92.1.7 that the K1 G4 (alone) and K2-SP G4s (together as a higher-order feature) have opposing roles in transcriptional regulation, with the former acting repressively and the latter acting to stimulate transcription. Further, they show that each G4 unit is significantly affected by the formation or disruption of the others, signifying G4-G4 crosstalk within a cellular context. The readout from each mutational state was more of a continuous distribution, rather than an all or nothing response. Overall, this study suggests that xG4s play a very nuanced role in governing transcriptional activation or repression. It remains to be seen whether a K2-SP disrupting small molecule, or SP-K1 stabilizing small molecule will act as an indirect c-Kit inhibitor.

## 3 Case study: targeting the hTERT core promoter xG4

**FIGURE 2:**
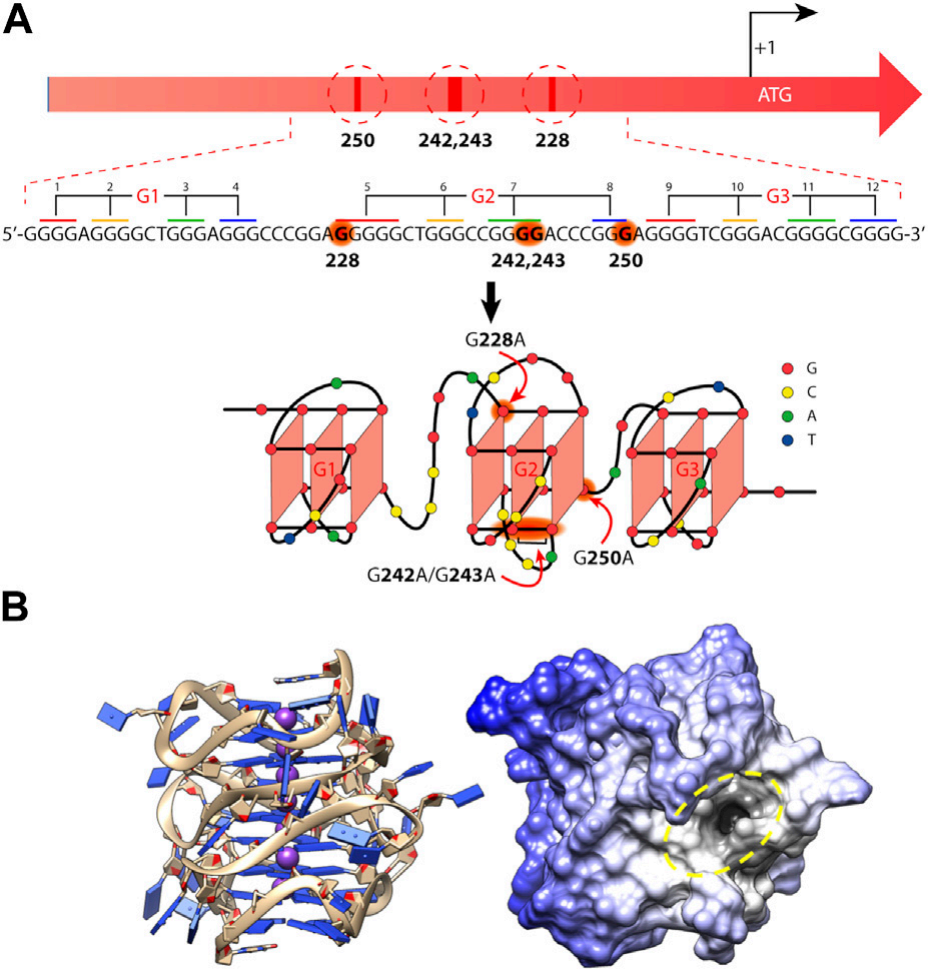
Mutations and folding of the hTERT core promoter G-quadruplex. **(A)** Cancer-specific mutations in the hTERT promoter’s primary structure that generate *de novo* binding sites for the ETS transcription factors and putative structure that was proposed for the G-rich (noncoding) strand of the hTERT promoter. Below is the sequence of the WT hTERT G4 motif and one conceivable way of G4 folding. The assignment of G-tracts (numbered) to three G4-motifs (G1, G2, and G3) is presented. Common mutation sites are shown in red in the hTERT G-rich sequence and in red arrows in the structural model. **(B)** The folded all-parallel hTERT xG4 represented with sugar-phosphate backbone as ribbons and nucleobases as slabs (left) and surface representation showing a large binding pocket (dark area inside of yellow dashed oval) at the junction between the first and second G4s. **(A)** was adapted from (Pavlova et al., 2022) and **(B)** was adapted from (Monsen and Trent, 2018).

One of the most studied xG4s to date is found within the *hTERT* core promoter region. *hTERT* encodes the protein catalytic subunit of telomerase, the ribonucleoprotein primarily responsible for maintaining telomere length homeostasis (Bryan and Cech, 1999). Although typically undetectable in somatic cells, hTERT is aberrantly over-expressed in more than 90% of aggressive cancers (Shay and Bacchetti, 1997), making it a long sought after cancer-specific target. The *hTERT* gene was first identified as harboring a putative xG4 in its promoter by Palumbo et al. (2009). The hTERT xG4 sequence consists of twelve G-tracts of three or more guanines that enable the maximum formation of three contiguous G4s (Figure 2).

Since its discovery, the tertiary structure of the *hTERT* xG4 has been intensely disputed. In the original work, Palumbo et al. showed by CD that the *hTERT* sequence was entirely parallel (Palumbo et al., 2009), although, based on their DMS footprinting results, they proposed a model consisting of a parallel G4 unit connected to an antiparallel G4 unit connected by a ∼26 nucleotide hairpin loop. About a year after, Micheli et al. (2010) independently proposed that the hTERT xG4 formed three parallel G4 units that were contiguous and stacked 3′ to 5′ based on CD and Taq polymerase stop assays. Later studies using an experimental small molecule and DMS footprinting assays reported yet another, slightly different, G4-hairpin arrangement (Song et al., 2019). However, structural studies of the sequence using a combination of CD, hydrodynamics, NMR, modeling, and small-angle X-ray scattering (SAXS) have since confirmed that the most consistent model is one in which three parallel G4s are tightly stacked in a stacked 3′ to 5′ arrangement (Figure 2) (Monsen et al., 2020). A recent DMS footprinting experiment confirms the all-parallel model as correct (Pavlova et al., 2022).

The *hTERT* xG4 is a potentially selective, transcriptionally repressive structure. The *hTERT* core promoter region has multiple non-coding mutations that are seen across many cancer types, with the two most prominent denoted “G228A” and “G250A”, that coincide with a robust increased telomerase activity (Killela et al., 2013). These mutations exist within G-tracts that form the central G4 unit of the three stacked all-parallel xG4 model (see Figure 2) (Micheli et al., 2010; Chaires et al., 2014; Monsen et al., 2020). Using luciferase expression assays of the wild type (WT) promoter versus mutant promoters of either G228A or G250A, Bell and colleagues showed that a robust increase in promoter activity is gained with either mutant (Bell et al., 2015). Further investigation showed that either G- > A mutation creates a *de novo* ETS (Erythroblast Transformation Specific) transcription factor consensus motif that is recognized by the transcription factor GABP (GA-binding protein). However, the creation of an ETS motif has been shown to be insufficient to fully explain the transcriptional changes seen in mutant cells (Kang et al., 2016).

Early investigations by Micheli et al. showed that the central G4, which encompasses either G- > A mutation, is unstable as an isolated monomeric G4, and only forms stably as a higher-order G4 assembly through stacking with both 5′ and 3′ G4 regions (Micheli et al., 2010). The destabilizing effects of the mutants on the overall xG4 have since been confirmed (Pavlova et al., 2022). A G4 ligand reported to refold and stabilize the hTERT xG4 WT structure (with or without G- > A mutations) was able to restore its repressive effects and showed good selectivity for the hTERT promoter over other genes with known promoter G4s (Kang et al., 2016). More recently, Monsen et al., (2022b) used a virtual screening approach to target the loop and G4-junctional regions of the all-parallel hTERT xG4 (Figure 2). Using a variety of *in vitro* binding and competition assays, the authors were able to find a drug-like small molecule that stabilized across the second and third G4 regions that showed high selectivity over duplex DNA and moderate selectivity over all other forms of DNA tested. In both cases, repression of hTERT expression was confirmed in breast cancer cell lines. Collectively, these studies reveal that the *hTERT* xG4 is an indirect target for down-regulating hTERT in cells. Further, these studies show for the first time that a higher-order promoter G4 can be targeted with selectivity using the unique features imparted from G4-G4 domains.

## 4 Discussion

Higher-order G-quadruplexes have emerged as selective targets in the promoters of thousands of annotated genes across the human genome. While xG4s offer some of the same protein binding recognition that monomeric G4s do, promoter xG4s also appear to encode for transcriptional status, possibly acting as titratable “dimmer switches” of gene activity. Structurally, xG4s offer a much richer drug targeting landscape consisting of G4-G4 interaction junctions adorned with protein-like binding pockets formed among sequence-specific loop features. This is emphasized by the decade-long pursuit targeting the *hTERT* core promoter xG4 which has resulted in both a unique receptor and the first drug-like small molecule targeting an xG4 with selectivity.

While xG4 studies to date have led to exciting new insight into their structural arrangements and potential regulatory mechanisms, there is still much to be understood. One of the major hurdles in studying xG4s is their recalcitrance to the traditional structural biology techniques NMR and X-ray diffraction. To date, all structural models have been derived from medium-to low-resolution integrative structural strategies and/or footprinting methods (Palumbo et al., 2009; Micheli et al., 2010; Rigo and Sissi, 2017; Monsen et al., 2020; Monsen et al., 2021; Monsen et al., 2022a; Pavlova et al., 2022). Cryo-EM is emerging as a possible solution to this problem (Monsen et al., 2023); however, atomic resolution of relatively small, potentially heterogeneous [e.g., G-tract isomers (Harkness and Mittermaier, 2017; Hennecker et al., 2022)] or flexible systems (e.g., inter-domain movements or long flexible loops) (Monsen et al., 2021; Monsen et al., 2022a; Monsen et al., 2023) is still a considerable challenge in the field (Herzik et al., 2019) [although scaffolds might offer a solution (Wu and Rapoport, 2021)]. A second major hurdle, as touched on above, is determining their biological mechanism. Molecular tools, such as xG4-specific fluorescent molecules (Summers et al., 2021) or antibodies (Biffi et al., 2013), should aid in revealing their spatial and temporal formation. Recently, the zinc finger-containing transcription factor Yin Yang-1 (YY1) was shown to bind G4s and bring two G4 domains into proximity through its dimerization (Li et al., 2021), offering a potential tool for investigating biological function of xG4s in a more biologically relevant context.
